# The Mysterious Noh Mask: Contribution of Multiple Facial Parts to the Recognition of Emotional Expressions

**DOI:** 10.1371/journal.pone.0050280

**Published:** 2012-11-21

**Authors:** Hiromitsu Miyata, Ritsuko Nishimura, Kazuo Okanoya, Nobuyuki Kawai

**Affiliations:** 1 Okanoya Emotional Information Project (OEIP), Exploratory Research for Advanced Technology (ERATO), Japan Science and Technology Agency (JST), Nagoya, Japan; 2 Okanoya Emotional Information Project (OEIP), Exploratory Research for Advanced Technology (ERATO), Japan Science and Technology Agency (JST), Wako, Japan; 3 Graduate School of Arts and Sciences, The University of Tokyo, Tokyo, Japan; 4 Brain Science Institute (BSI), RIKEN, Wako, Japan; 5 Graduate School of Information Science, Nagoya University, Nagoya, Japan; University of Sydney, Australia

## Abstract

**Background:**

A Noh mask worn by expert actors when performing on a Japanese traditional Noh drama is suggested to convey countless different facial expressions according to different angles of head/body orientation. The present study addressed the question of how different facial parts of a Noh mask, including the eyebrows, the eyes, and the mouth, may contribute to different emotional expressions. Both experimental situations of active creation and passive recognition of emotional facial expressions were introduced.

**Methodology/Principal Findings:**

In Experiment 1, participants either created happy or sad facial expressions, or imitated a face that looked up or down, by actively changing each facial part of a Noh mask image presented on a computer screen. For an upward tilted mask, the eyebrows and the mouth shared common features with sad expressions, whereas the eyes with happy expressions. This contingency tended to be reversed for a downward tilted mask. Experiment 2 further examined which facial parts of a Noh mask are crucial in determining emotional expressions. Participants were exposed to the synthesized Noh mask images with different facial parts expressing different emotions. Results clearly revealed that participants primarily used the shape of the mouth in judging emotions. The facial images having the mouth of an upward/downward tilted Noh mask strongly tended to be evaluated as sad/happy, respectively.

**Conclusions/Significance:**

The results suggest that Noh masks express chimeric emotional patterns, with different facial parts conveying different emotions This appears consistent with the principles of Noh which highly appreciate subtle and composite emotional expressions, as well as with the mysterious facial expressions observed in Western art. It was further demonstrated that the mouth serves as a diagnostic feature in characterizing the emotional expressions. This indicates the superiority of biologically-driven factors over the traditionally formulated performing styles when evaluating the emotions of the Noh masks.

## Introduction

Noh refers to a major form of Japanese traditional musical drama performed since the 14th century, i.e., the latter half of the Kamakura period in Japan’s history (for an overview, see [1]–[2]). It is characterized by extremely symbolized conventional performing styles. A Noh mask worn by skilled actors during performance is a hard wooden mask having fixed properties of facial components. At first glance, it often appears expressionless or mysterious, with specific emotions difficult to be attributed. However, the Noh dramas nevertheless involve multiple scenes in which various emotions of the characters are expressed. Instead of bearing no expressions, the Noh mask can be regarded to potentially convey all kinds of different emotions, i.e., “*mugen hyojo* (infinite facial expressions)”, according to different orientations of the head as well as the body [3]–[4]. Specifically, in the convention of the Noh drama, the mask is turned upwards when signifying a happy state, a gesture known as *terasu* (shining). By contrast, the mask is turned downwards when signifying a sad state, known as *kumorasu* (clouding) [3].

The question posited here is whether observers recognize emotional expressions in a Noh mask in the same ways as the rules of the drama intend to convey. Lyons et al. [5] examined how British and Japanese participants perceive facial expressions in a Noh mask shown at systematically different angles of inclination. Participants from both cultural groups tended to attribute happiness to a downward tilted mask, and sadness to a upward tilted mask. This trend remained consistent both when the facial image of chin and upper head contour was cropped, and when human face images were used instead of Noh mask images. These data were opposite to the conventional styles of the Noh drama. The facial action coding system (FACS), developed by Ekman and Friesen [6], was applied to interpret these results. Specifically, the downward tilted mask suggested raised cheek and pulled lip corner, which are elements of a happy expression. By contrast, the sad expression of the upward tilted mask was suggested to be mainly conveyed by raised inner brow and depressed lip corner. These interpretations were also in agreement with Kappas et al. [7], suggesting that vertical viewing angles alter the appearance of facial parts. For example, lip corners seen from above and below appear to be drawn upwards (as if being happy) and downwards (as if being sad), respectively. Minoshita et al. [8] also confirmed these findings by analyzing larger numbers of emotional categories. For example, downward tilted masks were recognized as happier, more composed, and less surprised, compared with upward tilted masks (see also [9]–[10]).

Nishimura et al. [4] further examined whether not only head inclination but also body postures of the *shite* (the main actor) may contribute to the viewers’ recognition of the emotional expressions. They used a downloaded succession of pictures that included either the face alone, or the body together with the face, while the actor was looking down to play a stylized sad act of a Noh drama. In both conditions, pictures with small downward inclinations tended to be recognized as relatively sadder, while those with larger inclinations as relatively happier. These trends were stronger for the pictures that included the whole body posture. Hiding the hand from the picture of the body failed to significantly alter these trends. Nishimura et al. [4] argued that emotions expressed by the whole body postures had stronger effects than those by a Noh mask alone, and that the actor’s initial movements may have determined the labels of emotions in both these conditions.

These preceding studies conducted so far are in agreement that inclination of the head as well as the body in the course of successive actions plays a significant role in the recognition of emotional expressions during Noh performance. The results, however, show trends opposite to the traditional rules of Noh [4]–[5]. To resolve these apparent contradictions, it should be beneficial to systematically examine how different facial parts contribute to the recognition of the emotional expressions, in either frontal or tilted pictures of Noh masks. For example, if certain part of a face consistently plays a primary role in the judgment of the emotional expressions, that facial part would turn out to be a biologically dominant factor in emotion recognition. As Ekman and Friesen [11] indicated, there could be emotional facial expressions that are biologically-based and are consistent across people from different cultures (see also [12]–[15]). By contrast, if the recognition of emotions consistently corresponds to the conventional rules of Noh, that would indicate the strong influence of historically formulated styles of the Noh drama on the recognition of emotion. In other words, detailed analyses of the viewers’ recognition of facial expressions and comparing them with the rules of Noh should help to elucidate the potential interactions between biologically-driven factors and culturally accumulated conventions in Japanese traditional art.

The purpose of the present research was to systematically examine how, and in what weight, different facial parts of a frontal or tilted Noh mask contribute to the recognition of different emotional expressions. Japanese participants were tested in both contexts of active creation (Experiment 1) and passive recognition (Experiment 2) of emotional facial expressions, using pictures of Noh masks presented on a computer display. Both experiments aimed to elucidate how facial components, including the eyebrows, the eyes and the mouth, may play significant roles in forming the happy or sad impressions of the mask as a whole.

## Results

### Experiment 1

**Figure 1:**
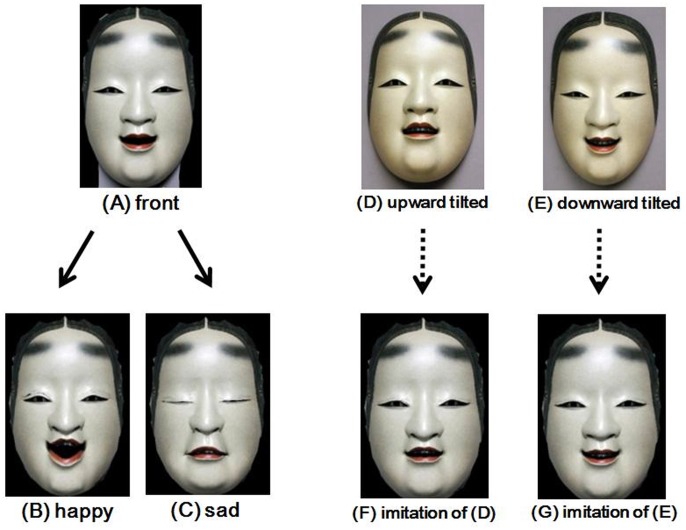
Examples of the Noh mask images in Experiment 1. (**A**) shows the frontal image of the Koomote mask presented at the beginning phase for all the four conditions. Participants changed facial parts of this image to create expressions according to the instructions given in each condition. (**B**) and (**C**) show the examples of faces that participants created in the happy and sad conditions, respectively. (**D**) and (**E**) show the upward tilted and downward tilted Koomote masks presented simultaneously with the frontal image in these conditions. (**F**) and (**G**) show the examples of faces that participants created by imitating (**D**) and (**E**), respectively.

For each of the four conditions, i.e., happy, sad, upward tilted, and downward tilted (**Figure 1**), **Table 1** summarizes rating values at each of the controllers averaged across participants. For each condition and facial action, one-sample *t*-tests with Bonferroni correction made comparisons with zero, to reveal the significantly manipulated controllers. Data for these conditions were then compared with each other, to examine which facial parts may share comparable changing patterns between happy/sad expressions and upward/downward tilted expressions. To describe these results, for the upward tilted condition, statistically significant components of facial actions such as raising the inner eyebrow (AU 1), depressing the lip corner (AU 15), and raising the chin (AU 17) corresponded to those observed for the sad condition. By contrast, raising the upper lid to wide open the eyes (AU5) for the upward tilted condition corresponded to that found for the happy condition. Data for the downward tilted condition overall showed trends opposite to those for the upward tilted condition, even though statistical support for this condition was relatively weak. Specifically, lack of lowering the outer brow (AUs 1 and 4), the trend to pull up the lip corner (AU 13), and lack of raising the chin (AU17) observed for the downward tilted condition corresponded to those for the happy condition. By contrast, the trend to make the eyes less open (AU 7) found for the downward tilted condition was parallel to that for the sad condition.

**Table 1:** Results of Experiment 1.

FACS name	AU	condition			
		happy	sad	upward tilted	downward tilted
Inner Brow Raiser	AU1	26.6 (7.7)	68.4*** (9.6)	30.3*** (3.9)	7.0 (4.1)
Outer Brow Raiser	AU2	14.7 (7.2)	0.0 (0.0)	0.1 (0.1)	9.0 (3.6)
Brow Lowerer	AU4	3.7 (2.0)	59.9** (10.5)	3.8 (2.2)	25.1 (8.9)
Upper Lid Raiser	AU5	45.6** (8.3)	22.4 (8.2)	24.6* (5.9)	13.5 (4.8)
Lid Tightener	AU7	4.9 (2.9)	17.2 (6.3)	1.2 (1.2)	13.1^+^ (3.7)
Eyes Closed	AU43	0.0 (0.0)	9.6 (3.3)	0.1 (0.1)	0.0 (0.0)
Cheek Raiser	AU6	60.8*** (8.2)	0.0 (0.0)	2.9 (1.4)	21.1^+^ (6.1)
Nose Wrinkler	AU9	0.6 (0.6)	18.5 (6.0)	4.3 (2.3)	1.2 (0.8)
Lip Corner Puller	AU12	72.3*** (8.2)	0.0 (0.0)	7.6 (6.9)	8.9 (3.9)
Sharp Lip Puller	AU13	45.4** (8.7)	1.6 (1.4)	6.0 (2.7)	17.5^+^ (4.6)
Lip Corner Depressor	AU15	0.0 (0.0)	100.0 (0.0)	31.4** (6.3)	9.8 (4.3)
Lip Stretcher	AU20	39.2** (7.7)	68.8*** (8.7)	5.0 (2.5)	6.2 (3.3)
Upper Lip Raiser	AU10	13.1 (7.2)	13.2 (5.0)	13.4 (4.7)	2.4 (1.8)
Lower Lip Depressor	AU16	36.1^+^ (10.0)	0.1 (0.1)	7.1 (3.8)	6.8 (2.5)
Lip Pucker	AU18	3.9 (3.7)	0.4 (0.4)	19.1* (4.9)	15.0 (5.3)
Lip Tightener	AU23	4.1 (3.0)	47.4* (10.3)	7.1 (4.0)	25.1** (4.8)
Lip Pressor	AU24	5.4 (5.2)	20.1^+^ (5.6)	5.6 (2.8)	7.4 (3.7)
Mouth Stretch	AU27	18.9 (6.9)	5.6 (4.0)	4.6 (2.2)	0.0 (0.0)
Lips Part	AU25	10.8 (4.2)	2.3 (1.7)	4.4 (2.1)	1.2 (1.2)
Jaw Drop	AU26	4.6 (3.3)	4.3 (3.2)	4.4 (3.3)	1.9 (1.4)
Chin Raiser	AU17	19.6 (9.2)	47.1* (10.4)	57.4*** (7.7)	10.1 (4.2)

Mean values of the controller bars on FaceTool software for the four conditions to make changes to the facial parts are indicated using FACS (facial action coding system) names, accompanied by AU (Action Unit) numbers. Standard errors of the mean are shown in parentheses. Asterisks indicate values significantly larger than zero. ^+^: *p*<0.10; *: *p*<0.05; **: *p*<0.01; ***: *p*<0.001.

**Figure 2:**
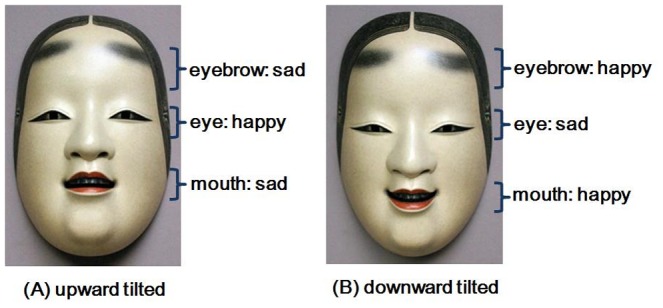
A schematic illustration summarizing the main results of Experiment 1. Depicted are the emotions represented differentially by each facial part of the upward tilted and downward tilted Noh masks.

To further illustrate the correspondence between these results, **Figure 2** depicts the emotions (happy or sad) expressed by each facial part of the upward tilted and downward tilted Noh masks. For the upward tilted Noh mask images, the eyebrows and the mouth turned out to have common features with those when creating sad expressions, whereas the eyes with those when making happy expressions (**Figure 2** **(A)**). This contingency overall tended to be reversed for the downward tilted Noh mask images (**Figure 2** **(B)**).

### Experiment 2

**Figure 3:**
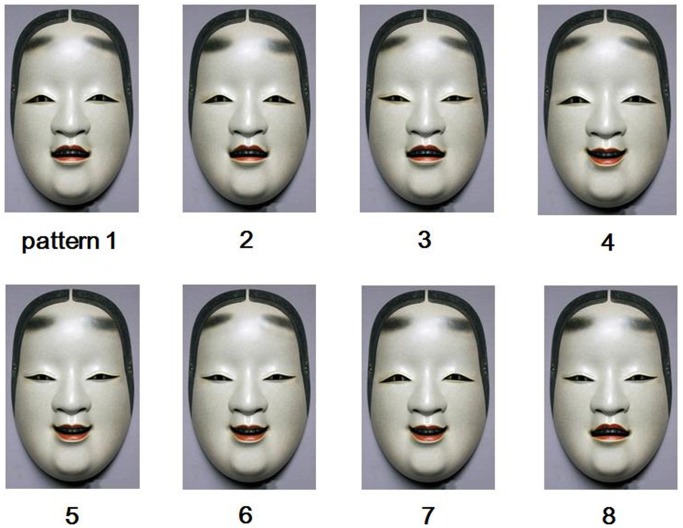
The eight synthesized facial patterns of the Noh mask used in Experiment 2.

For each synthesized image (**Figures 3** **and** **4**), the proportion of the “happy” evaluation was determined based on the number of times with the “happy” evaluation out of the four repeated trials. Because the data points were given in the proportions, the data were transformed for the statistical analyses, using the equation *r’* = arcsin (√*r*), to make distributions more normal. Whereas the binomial choices are at the nominal level of measurement, this transformation allows treatment of the data at the interval scale [5], [16]. For these choice proportions, a one-way repeated measures analysis of variance (ANOVA) with facial pattern (8 patterns) as a within-subject factor was conducted. **Figure 5** shows the mean proportions of “happy” evaluations for each pattern. The main effect of the facial pattern turned out to be statistically significant (*F* [7,434] = 445.5, *p*<0.001). Multiple comparisons with Bonferroni correction revealed that there were statistically significant differences between all the 16 combinations of patterns 1, 2, 3, 8 and patterns 4, 5, 6, 7 (all *ps*<0.001). By contrast, no statistically significant differences were found between any combinations of patterns within patterns 1, 2, 3, 8 (all *ps*>0. 308), nor within patterns 4, 5, 6, 7 (all *ps*>0.143). Also, one-sample *t*-tests with Bonferroni correction compared the proportion of “happy” evaluations for each facial pattern with the chance level (i.e., 50%). For patterns 4, 5, 6, and 7, the proportions were significantly higher than the chance level (mean = 95.4%; *t* = 18.068–25.735, all *ps*<0.001, corrected), whereas for patterns 1, 2, 3, and 8, the proportions were significantly lower than the chance level (mean = 12.1%; *t* = −19.458 – −8.926, all *ps*<0.001, corrected). Seeing these data in light of the emotions expressed by each facial part shown in **Table 2** (and **Figure 3**), the choice proportions best correspond to the emotions expressed by the mouth, rather than to the other facial parts or combinations of multiple facial parts. These results thus show that, irrespective of which eyebrows or eyes used, the synthesized images having the mouth of the upward tilted Noh mask image strongly tended to be evaluated as sad, whereas those having the mouth of the downward tilted Noh mask image strongly tended to be evaluated as happy.

**Figure 4:**
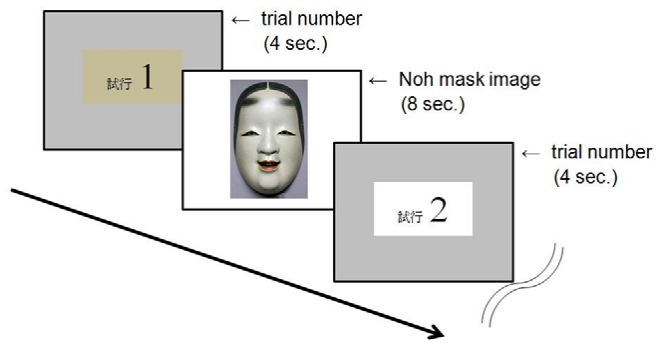
Diagram depicting examples of trials in the test session of Experiment 2. The letters on the first and third displays indicate “Trial 1” and “Trial 2”, respectively.

**Figure 5:**
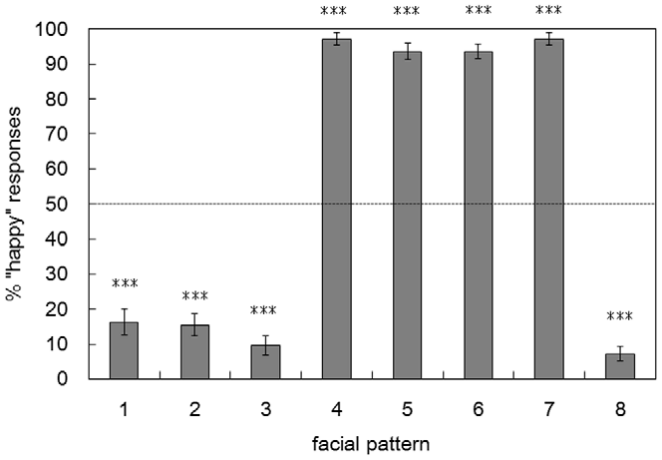
Proportion of the “happy” evaluation for each synthesized facial pattern in Experiment 2. The dashed horizontal line indicates the chance proportion of the “happy” responses (i.e., 50%). Error bars indicate standard errors of the mean. Asterisks indicate statistically significant differences from the chance level (***: *p*<0.001).

**Table 2:** Description of the eight facial patterns of the synthesized Noh mask images used in Experiment 2.

	facial part	facial pattern							
		1	2	3	4	5	6	7	8
head inclination	eyebrows	upward	downward	upward	upward	downward	upward	downward	downward
	eyes	upward	upward	downward	upward	downward	downward	upward	downward
	mouth	upward	upward	upward	downward	downward	downward	downward	upward
emotion expressed	eyebrows	sad	happy	sad	sad	happy	sad	happy	happy
	eyes	happy	happy	sad	happy	sad	sad	happy	sad
	mouth	sad	sad	sad	happy	happy	happy	happy	sad

The upper half of the table describes head inclination of the images from which each facial part was cropped (upward tilted or downward tilted). For example, pattern 1 shows that the eyebrows, the eyes, and the mouth had all been synthesized using those of the upward tilted Noh mask image. The lower half of the table describes the emotions that are deemed to be expressed by each facial part of the upward and downward tilted Noh mask images (happy or sad), based on the results of Experiment 1.

**Figure 6:**
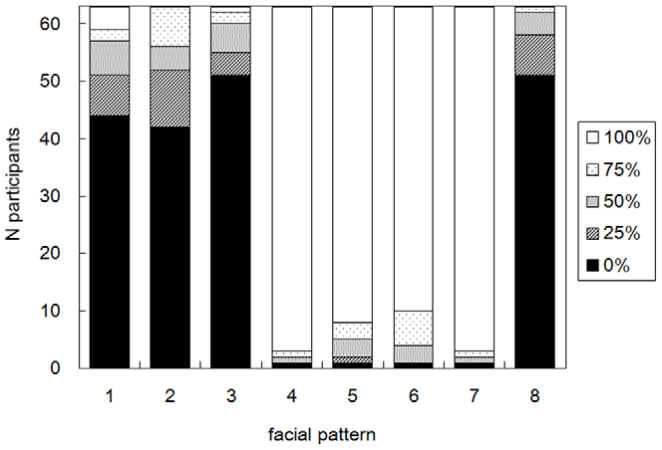
Numbers of participants for each proportion of “happy” evaluation in Experiment 2, shown for each synthesized facial pattern. “100%” in this graph means that the facial pattern was evaluated as “happy” for all the four repeated presentations, whereas “0%” means that the pattern was evaluated as “sad” for all the four repetitions.

In addition, **Figure 6** depicts numbers of participants for each proportion of the “happy” evaluations (i.e., 0%, 25%, 50%, 75%, and 100%). As apparent from the graph, for patterns 1, 2, 3, and 8, the largest numbers of participants evaluated the expressions as “sad” for all the four repeated trials (1/5 binomial tests; all *ps*<0.001). In contrast, for patterns 4, 5, 6, and 7, the largest numbers of participants evaluated the expressions as “happy” for all the four repetitions (1/5 binomial tests; all *ps*<0.001). Seeing these data again in light of **Table 2** and **Figure 3**, the results further show that the participants’ evaluation of the emotional expressions was distinctively made based on the shape of the mouth.

## Discussion

The present research examined the roles of different facial components in characterizing the emotional expressions of the Japanese Noh masks, in both contexts of active creation of facial expressions and passive emotion recognition of the synthesized facial patterns. In Experiment 1, we presented upward tilted and downward tilted Noh masks, which each corresponded to the stylistic gesture signifying happiness (*terasu*) and sadness (*kumorasu*). In these Noh mask images, the eyebrows, the eyes, and the mouth each expressed different emotion, forming composite expressions involving both happiness and sadness simultaneously. In Experiment 2, when presented with the Noh mask images of which the eyebrows, the eyes, and the mouth each expressed different emotion, participants primarily used the shape of the mouth in differentiating the emotions. It could thus be proposed that the mouth serves as a diagnostic feature in the categorical judgment of emotions when viewing the Noh masks. Taken together, these results suggest that Noh masks express chimeric emotional patterns that changes according to the actor’s head orientations, and that among different facial parts the mouth serves as the most distinctive feature in determining the overall emotional categories.

The major question posed in the Introduction was how biologically-driven and cultural/conventional factors may play dominant roles when recognizing emotions of Noh masks. With regard to this question, the results of Experiment 2 seem most convincing. They clearly demonstrated that the judgment of emotional expressions of a Noh mask is predominantly made based on the shapes of the mouth, with the mouth of an upward/downward tilted Noh mask expressing sadness/happiness, respectively. Thus, when presented with the face of a Noh mask alone, the categorization of the emotional expressions seems to rely primarily on a biological feature such as the shape of the mouth, rather than on the traditionally formulated styles of the Noh drama. This further seems in agreement with the notion that the recognition of facial expressions is universal and thus is biologically-based [11]–[15], even when the artificial masks are used instead of the human faces.

Such importance of the mouth could be considered in light of the literature on facial recognition. Traditionally, Dunlap [17] reported evidence of the superiority of the mouth in the recognition of emotions (see also [18]). More recent reports further focus on the cultural differences in the importance of different facial parts in recognizing emotions. Yuki et al. [19] presented emoticons to both Americans and Japanese, and found that Americans located expressions at the mouth, recognizing :) as happy and :(as sad, while Japanese found them in the eyes, seeing ^Λ^_^Λ^ as joyful and ;_; as tearful. Jack et al. [20] analyzed eye movements of both Western and Eastern observers when recognizing emotional facial expressions, and found that Western observers distributed their fixations evenly across the face including the mouth, whereas Eastern observers persistently fixated the eye region while rarely gazing at the mouth. In a consistent way, Ozono et al. [21] showed that Americans focused on the lower half of a face when determining its trustworthiness, whereas Japanese emphasized the upper half. Despite these suggested cultural differences, our data involving Japanese participants and Japanese traditional art strongly supported the importance of the mouth [17]–[18]. This may further favor the strong influence of biological factors in emotion recognition of Noh masks. Future studies involving participants from different cultures and different types of masks may confirm these possibilities.

The overall trends of the present findings also seem consistent with those from the preceding studies using tilted Noh masks [4]–[5]. That is, these studies revealed that Noh masks tilted upwards tend to be recognized as sad, whereas those tilted downwards tend to be recognized as happy, as opposed to the traditional styles of the Noh drama. It seems worthwhile considering the potential factors that consistently cause these opposite trends. Regarding the present study, participants did not know the Noh rules *terasu*/*kumorasu*, suggesting that the observed trends may not directly reflect their cultural knowledge. The Noh drama has many stylistic rules other than those regarding the Noh masks, including inclination and successive movements of the actor’s body as well as the background chorus and instrumental music [3]. Observers take in all these information to appreciate the intended emotions. Consequently, during the actual performance of the Noh drama, these multiple conventional rules may holistically play a major role in the appreciation of emotions. For example, Nishimura et al. [4] proposed that the initial movements of the stylized actions, rather than the later ones, may determine the category of the emotions for that whole action. This was suggested to explain why the intended emotions are appreciated appropriately in the Noh drama, even if the stylistic rules sometimes appear to contradict with the biological mechanisms of emotion recognition. Thus, a plausible scenario could be that the dominance of biological factors observed when presented with the Noh masks alone is taken over by the cultural/conventional factors when the drama as a whole is involved. To elucidate the effects of such conventional factors, it would be beneficial to systematically manipulate information conveyed in the drama, such as the movements of multiple actors and/or background music.

The notion that Noh masks express chimeric and composite emotional patterns, as suggested in Experiment 1, further leads to the assumption that the masks with any given head inclinations possess different composite expressions, each of which involves multiple emotions at a time. Such mysterious qualities seem to go in line with what is observed in the Western art. For example, Mona Lisa is suggested to change its expressions depending on which facial parts the observer looks at, thereby possessing an elusive quality in it [22]. Bohrn et al. [23] simulated this phenomenon using non-Mona-Lisa facial stimuli, by presenting a smiling mouth when the viewer looked at the eyes, and a neutral mouth when the viewer gazed at the mouth. The faces in this Mona-Lisa condition were evaluated as more attractive and trustworthy than the neutral faces, even though the viewers’ confidence on their own ratings were the lowest in the Mona-Lisa condition. Similar mysterious facial expressions are observed not only in Renaissance paintings but also more widely in Western art [24]. With regard to the Japanese tradition of the Noh drama, “*yugen*”, or subtle profundity, is deemed to be the highest aesthetic principle [25]. Consequently, subtle and composite emotional expressions are more highly appreciated than the expression of a single pure emotion in the Noh drama. The present findings thus seem consistent with the potential of a Noh mask to convey countless different emotions (e.g., “*mugen hyojo*” as termed in the Introduction), despite its fixed physical properties.

To summarize, the present study primarily suggested the dominance of biological factors such as the shape of the mouth when evaluating the emotions of Noh masks. They further proposed the potential influence by the conventional performing styles if the Noh drama as a whole is involved, as well as implications for an even higher aesthetic state such as *yugen*. In relation to these issues, the expertise of the participants to the Noh drama would be one important challenge for the future quest. That is, the present participants were naïve in terms of the performing skills of Noh, despite their basic knowledge about Noh. This may have led to the dominance of the biological factors in emotion recognition. In the course of the professional expertise, the trainees should learn the determined associations between certain head/body postures and corresponding emotional expressions. Thus, it might be plausible that highly skilled Noh actors tend to evaluate upward tilted Noh masks as happy (*terasu*) and downward tilted masks as sad (*kumorasu*), in accordance with the rules of Noh. These experts, compared with novices, may also have a more detailed and elaborate cognitive sketch on how different facial parts convey subtle emotional qualities. Also, it would be promising to include participants truly naïve to the Noh drama, such as those from North America or Europe, to examine the potential effect of cultural background including basic knowledge about Noh. Methodologically, the two-alternative forced choice procedure used in Experiment 2 seems effective so long as the study focuses on the categorical judgment of emotions. The promising alternatives, however, could be to use continuous scales or choice procedures with more options, which should provide stronger statistical power. It would also be beneficial to include a control condition using humans’ facial images, to examine similarities and differences between Noh masks and human faces. Actually, Lyons et al. [5] found that the decline of “happy” responses at large downward tilting angles for Japanese viewers was present only for Noh masks, not for human faces. Further investigations from these various perspectives should help to further elucidate how biologically-driven factors as well as conventional styles may influence the recognition of emotional information to attain higher aesthetic qualities in traditional performing art.

## Materials and Methods

### Experiment 1

The specific purpose of the first experiment was to examine to what extent facial expressions of an upward or downward tilted Noh mask shared common features with faces that are typically recognized as happy or sad. Participants actively changed facial expressions of a Noh mask image presented on a computer screen by moving each facial part, and either created happy or sad expressions, or imitated an upward or downward tilted Noh mask. Based on previous findings [4]–[5], we hypothesized that happy faces would have many common features with those imitating downward tilted faces, whereas sad faces with those imitating upward tilted faces.

#### Participants

Fourteen healthy Japanese adults (7 females and 7 males; age, 20–23 years; mean age = 21.4 years, *SD* = 1.0) participated. All participants in both experiments were university undergraduate students recruited at Nagoya, Japan, who were exposed to Western culture in comparable ways as Japanese people in general are. All participants in both experiments were familiar with Noh masks, either as images or objects, but had not received specialized training with Noh prior to participation. A preliminary enquiry had revealed that the same population of participants was ignorant of the Noh gestures *terasu*/*kumorasu* to signify happiness/sadness. All participants had normal or corrected-to-normal vision. The study was approved by the Ethics Committee of the Japan Science and Technology Agency. All participants from both experiments gave written informed consent upon agreement cooperate. They were not compensated for participation.

#### Stimuli

A frontal image of a Koomote mask was used for the beginning phase of the experiment (**Figure 1**). The mask had been carved by Akira Kurabayashi, a Japanese professional Noh mask artist. Koomote is a mask that represents a cute young girl below 20 years of age [26]. The size of the image presented was 11.4 degrees wide and 18.5 degrees long in terms of in visual angle. For the upward tilted and downward tilted conditions (see the Procedure section for details), also used were the images of the same Koomote mask that had been photographed at the vertical viewing angles of 10 degrees above (upward tilted) or below (downward tilted) front. These tilted images intended to show the examples of the expression of joy (*terasu*) and sorrow (*kumorasu*) in the conventional rules of Noh. All these images were copy-free and were downloaded from the artist’s website [27].

#### Procedure

FaceTool, a software for creating facial expressions, was used. The software had been developed by the laboratory of Professor Hiroshi Harashima, The University of Tokyo, Tokyo, Japan [28]. Using FaceTool, one can actively change the shapes of different facial parts, including the eyebrows, the eyes, the cheeks, the month, and so on, to create various facial expressions. The software had 23 horizontal bars as controllers, each of which labeled “eyebrow: inner brow raise”, “lip: lower lip depress”, and so on, which were used to manipulate shapes of each facial part. These actions of facial parts corresponded to the Action Unit (AU) used in FACS [6], a system that enables description of humans’ facial expressions based on the movements of mimetic muscles.

The experiment took place in a room dim, sound-attenuated room equipped with a personal computer. FaceTool software and the test stimuli were presented on a 48 cm (19.0 inches) flat-panel, LCD monitor (FlexScan S1932, EIZO, Ishikawa, Japan), whose dot response was deemed very fast. The display was located on a table in front of the participant, who sat on a comfortable chair. The distance between the monitor and the participant’s eyes was set at 57 cm, so that one centimeter on the screen corresponded to a visual angle of approximately one degree. By manipulating the controllers on FaceTool, participants changed the frontal image of the Noh mask to four different facial expressions: happy, sad, upward tilted, and downward tilted (**Figure 1**). Because all the participants were Japanese, the Japanese terms *“yorokobi”*, *“kanashimi”*, *“ue-muki”*, and *“shita-muki”* were used to refer to these conditions, respectively. Within-subject design was used, so that all participants were exposed to these four conditions. Each condition was presented once, and the order was counterbalanced across participants. For the happy and sad conditions, participants were instructed to change the image so that they would feel that the face typically represents these emotions, while referring to no other facial images. For the upward tilted and downward tilted conditions, participants were told to change the frontal image of the mask by imitating an upward or downward tilted Koomote image that was simultaneously presented to the left of the frontal image. Changes to the facial components were made by scrolling the indicator at each controller from left to right, based on the extent to which each action was true for the intended facial expression. The rating value for each controller was given on a scale of 0 to 100. For all the participants and conditions, rating values at all the controllers were recorded. There was no limited time before participants completed each condition, which was terminated when participants signaled that they had finished creating the expression as instructed. The duration for the whole experiment accounted for approximately 30 minutes.

### Experiment 2

The results of Experiment 1 suggested that the upward and downward tilted Noh masks are a so-called chimera, in that different facial parts express different emotions. Based on these findings, the next question concerned how viewers may weight emotions expressed by different facial parts, when presented with a face each part of which expressing different emotions. If viewers use any certain facial part predominantly to judge the emotions of the whole face, then their evaluations would show distinctive patterns according to the shape of that facial part. Otherwise, if certain combinations of multiple facial parts play a more crucial role, then evaluations would exhibit more complex patterns. Experiment 2 introduced the context of passive facial recognition on a computer screen. Participants evaluated the emotions of the synthesized Noh mask images, of which the eyebrows, the eyes, and the mouth expressed different emotions, respectively.

#### Participants

Sixty-three healthy Japanese adults (29 females and 34 males; age, 18–22 years; mean age = 19.1 years, *SD* = 0.7), other than those included in Experiment 1, participated.

#### Stimuli

Frontal, upward tilted, and downward tilted images of the Koomtoe mask carved by Akira Kurabayashi, the identical ones as those used in Experiment 1 (**Figures 1** **(A)**, **(D)**, and **(E)**, respectively), were modified with Adobe Photoshop CS4, to create stimuli for Experiment 2. The facial areas involving the eyebrows, the eyes, and the mouth were each cropped from the upward tilted and downward tilted Noh mask images. These different areas were then overlaid to the frontal Noh mask image, so that the eyebrows, the eye and the mouth would each express different emotions. Because there were eight combinations of the facial parts, there were eight different synthesized facial patterns (**Figure 3**; see **Table 2** for description of these patterns). The size of all these stimuli were 13.7 degrees wide and 22.6 degrees long in in terms of visual angle.

#### Procedure

The experiment took place in a room with a personal computer equipped for each participant. All participants were instructed and tested at a time. The test stimuli were presented on a 43 cm (17.0 inches) TFT LCD monitor (VL-17SE, Fujitsu, Kawasaki, Japan) located on a table in front of the participant, who sat on a comfortable chair. The distance between the monitor and the participant’s eyes was set at approximately 50 cm. The test stimuli were presented using Microsoft PowerPoint 2007. Before the main test session, there were three practice trials using images other than those for the test, for instruction of the general procedure. During the test session that followed, each of the eight different patterns of the synthesized Noh mask images was presented four times, thereby 32 trials forming the session. The order of these patterns presented was pseudo-randomized for each participant. **Figure 4** shows the flow of the typical trials for the test session. For each trial, the number of that trial first appeared for four seconds, followed by the synthesized Noh mask image for eight seconds at the center of the display on a white background. The duration of the presentation of stimuli was controlled by a personal computer, and the displays switched automatically following the determined durations, regardless of the participants’ responses. Participants were informed during the instruction that they would not be allowed to terminate the session on the halfway. Participants answered whether each displayed Noh mask image expressed happy or sad emotions (using the Japanese terms “*yorokobi*” or “*kanashimi*”), by filling in the evaluation sheet placed on a table in front of them. Following Lyons et al. [5], the answer had to be given in the form of a two-alternative forced choice, and thus no answers in between were allowed. Use of a comparable procedure enables effective comparisons between the preceding and the present studies. More recent studies on emotional facial expressions also employed this procedure [29], [30]. The duration of the test session was approximately seven minutes, and the whole experiment lasted for approximately 20 minutes.
